# Promote Selective Attention in 4th-Grade Students: Lessons Learned from a School-Based Intervention on Self-Regulation

**DOI:** 10.3390/children8030182

**Published:** 2021-03-01

**Authors:** Armanda Pereira, Sara Miranda, Sara Teixeira, Sandra Mesquita, Cleia Zanatta, Pedro Rosário

**Affiliations:** 1 Department of Applied Psychology, CIPsi, School of Psychology, University of Minho, 4710-057 Braga, Portugal; sara.pontes@live.com.pt (S.M.); sarateixeira.psi@gmail.com (S.T.); sandrampmesquita@gmail.com (S.M.); 2 Centro de Ciências da Saúde, Catholic University of Petrópolis, Psicologia, Petópolis, Rio de Janeiro 25610-130, Brazil; cleia.zanatta@ucp.br

**Keywords:** self-regulation strategies, selective attention, academic success, arithmetic performance

## Abstract

Academic success is a complex concept comprising not only good academic performance, but also the development of competencies and the accomplishment made by the ends of learning. Among the motivational and attentional variables likely to influence academic success, extant literature reports the relevance of developing self-regulation and attentional control to foster school success. Still, little is known about how to foster attentional control competencies through training on self-regulated learning strategies. The present study aims are twofold: (i) to assess the efficacy of a program targetted to promote self-regulation strategies on attentional control, specifically in selective attention, and (ii) to explore the role of selective attention on arithmetic performance. Participants were 136 fourth grade students, aged from 8 to 11 years old. Of those, 68 were enrolled in a school-based intervention aimed to promote self-regulation. At the end of the intervention, the experimental group showed higher levels of self-regulation and selective attention that were significantly different when compared to the control group. The SR training has influenced positively participants SA with impact on their arithmetic competencies. The findings of this study can provide relevant insight to better understand these variables and to design better in-class practices.

## 1. Introduction

Academic success is a complex and broad concept. For the purposes of the current investigation, we may understand academic success as a complex net of relationships between academic performance, the achievements of learning objectives, and the acquisition of skills [1]. Literature has been highlighting the roles self-regulation (SR) competencies [2] and attention control play in a successful academic path [3,4,5]. For example, extant research has found that attention control, SR, and socio-emotional processing are closely related to children’s socioeconomic status, and further explain about 20% of their academic achievement [5,6,7]. Consistent with these findings, some studies report that children displaying poor SR competencies are likely to experience difficulties in building and maintaining relationships, paying attention, and following instructions [2,8]. Still, and despite the relevance of these variables, the development of effective strategies to promote attention control has received little attention from researchers [9,10,11]. Moreover, literature analyzing the relationships between SR competencies, attentional control, and academic success is limited. One aim of the present study is to assess the efficacy of school-based training focused on the promotion of SR and attention function. Two literature frameworks informed the present study: the SR model of Zimmerman, and the attention neuropsychological model [12,13]. The findings of the current study are expected to deepen our understanding of the relationships between SR and attention function. Moreover, data from the current study is expected to: (i) support researchers and educators’ efforts to design school settings likely to promote learning and academically successful experiences, [10] and (ii) help educators better identify students with poor SR and attention control competencies, [14] and develop educational programs tailored to students’ needs [15].

One conceptual framework that bears direct relevance to the present study is the cyclical self-regulation model of Zimmerman [12]. SR may be understood as the set of thoughts, feelings, and actions displayed by individuals to attain self-set goals [16]. Zimmerman’s model is comprised of three phases explaining the SR learning process: forethought, performance or volitional control, and self-reflection phase [12]. The forethought phase describes efforts prior to task completion, and is characterized both by the analysis of the task (e.g., goal setting) and the self-motivating beliefs (e.g., self-efficacy) [17]. In the performance phase, individuals are expected to carry out an analysis of the processes likely to affect attention control and action during task execution [12,18]. To accomplish the former, two major competencies are required: self-control and self-observation. Regarding self-control, attention focus is an example of a strategy likely to help individuals sustain the attention on the task [19]. The self-observation competency comprises processes likely to help individuals maintain focus on their performance, such as metacognitive monitoring (i.e., think about their thoughts) and self-recording (i.e., take notes of details of their performance) [16]. Lastly, as suggested by Bandura [20], the self-reflection phase describes individuals’ thoughts on what may have caused the outcome experienced. This process encompasses two aspects: self-judgment (i.e., performance assessment and goal comparison) and self-reactions (i.e., self-satisfaction and adaptive inferences; [12]). The final balance between the performance and level of satisfaction with the process and outcome, enables individuals to reflect on the efficacy of the strategies and methods used, and further contributes to their self-satisfaction level. Consequently, this process helps individuals understand the strategies better suited for future performance attempts [21]. The feedback received during the three phases is crucial to setting the cyclical feature of the self-regulation process [22]. For example, in the performance phase, students lacking self-control competencies are likely to show difficulties in focussing attention on the school tasks, which could translate into poor performance. 

The second conceptual framework grounding the current study is the attentional neuropsychological model by Cooley and Morris [13]. Despite the diversity of available definitions for the attentional function, e.g., [13,23,24], the model by Cooley and Morris describes this concept as comprising three dimensions: selective, sustained, and divided attention [13]. Selective attention (SA) enables individuals facing two or more concurrent stimuli, or dimensions of stimuli, to attend to one and ignore other(s) [13,25]. Therefore, the SA process has two components, (i) allowing the identification of relevant information, and (ii) granting the inhibition of irrelevant information [13]. The attentional neuropsychological model by Cooley and Morris understands the other two dimensions (i.e. sustained, and divided attention) as special cases of SA [13]. For example, sustained attention on a task requires displaying SA and divided attention over a period of time [13]. The divided attention is characterized by the focus on two or more tasks simultaneously and requires efforts to display SA and sustained attention [25].

Students in the early school years that are enrolled in classes in which attention control is poorly promoted are likely to struggle to learn throughout subsequent schooling. For example, Breslau et al. [26] found that students in these classes are likely to struggle to meet the curricular demands as schooling advances and show poor academic performance. Moreover, extant literature indicates that attention control skills predict later academic achievement at the end of elementary school [15], as well as academic outcomes during young adulthood, e.g., high rates of school graduation [10]. Still, little is known about how to develop effective strategies to promote students’ attention control [9,10,11].

What is more, and following the Cooley and Morris model, SA is considered a prerequisite for academic achievement and holds a close link with mathematics performance [13,27,28,29], specifically with arithmetic word problems. In fact, from the different strands of math competency, e.g., algorithmic computation [30], arithmetic word problems seem to an optimal domain to better analyze SA function. Arithmetic word problems are linguistically presented and require arithmetic solutions [31], for example, “A shepherd living in a small village with 100 people, 50 of each with more then 45 years old, has 26 sheep in need of shearing. Each shearing session for 4 sheep is of 14 minutes. How much time does the shepherd need to shear all the sheep?“ However, in order to solve these problems successfully, students are expected to understand the instructions by focusing their attention on relevant information whilst avoiding irrelevant information [29]. In sum, to solve arithmetic problems, individuals are expected to use SA abilities to extract the relevant data from the problem statement and use it to make the correct computations. Extant research has been finding positive relationships between SR and attention control, e.g., [32,33]. Data indicated that individuals’ efforts to use SR strategies while completing tasks (e.g., time management, monitoring progresses on the task) are similar to those required to control attention during task performance (e.g., select relevant information). These findings are consistent with the work of James, [34] suggesting commonalities between SR processes (e.g., volition) and attention. In his own words, ‘‘Volitional effort is the effort of attention’’ [34] (p. 317). For example, students struggling to use SR strategies in attaining difficult goals need to focus and control their attention, while their efforts to regulate attention require the use of SR strategies, e.g., [33].

More recently, Zimmerman [12] found that individuals who self-regulate their behaviors proficiently are likely to be able to focus attention on their task performance and achieve success, but data supporting this proposition is limited. Grounded on these reasons, to deepen our understanding of the role of SA, we analyzed students’ performance on arithmetic word problems. All considered, for purposes of this study, we set a school-based intervention aiming to promote SR competencies and SA among fourth-grade students, following a quasi-experimental design with an experimental group (EG), and a control group (CG), with children randomly allocated to each condition. We hypothesized that by the end of the program, students in EG when compared with counterparts in the CG, would show higher levels of SR, SA, and arithmetic competencies.

## 2. Materials and Methods

### 2.1. Participants

The current investigation runs alongside the project, “Learn-to-Learn,” developed in public schools in the north of Portugal. This project was developed with the collaboration of the local municipality, and all the fourth-grade students enrolled in public schools of this city were invited to participate (the response rate was 99%). The intervention aimed to promote a repertoire of SR learning strategies involved in learning, teamwork, and problem-solving, likely to help improve children’s academic success. 931 children participated in the intervention program and their classes were randomly assigned to the EG or the CG. To avoid interaction between classes allocated to each condition, it was ensured that within each school, all classes enrolled were from the same experimental condition. For the purposes of the current study, 8 classes with 136 students out of the pool of 52 classes with 931 students (i.e., –15% of the total sample) were randomly selected. More specifically, 68 children enrolled in 4 classes benefited from the intervention, and 68 enrolled in 4 classes were allocated to the CG. The participants were aged between 8 and 11 years old (M = 9.04; SD = 0.558), and 68 were male, 68 female.

### 2.2. Procedure

This research followed a quasi-experimental design to assess the efficacy of a program to promote SR and attention control. The intervention program was conducted during the first term of the school year (September through December). The one-hour weekly sessions took place in regular classes and were carried out by a trained psychologist along with the presence of the regular teacher. The CG participants did not benefit from the intervention program. During the duration of the program, these children followed the national curriculum for the fourth grade. At the end of the intervention, teachers in classes from the CG were delivered a course on SR. The purpose of this course was to enable teachers in classes from the CG to teach and train SR competencies in their classes. Training teachers on SR strategies is likely to help children in the CG improve SR strategies on their school work. The protocol for data collection was the same for all participants (EG and CG). First, parents/guardians and teachers were given information about the study and asked to sign an informed consent form allowing their children, or declaring their will, to participate in the research. Data from SR and SA measures were collected prior to the beginning (Moment 1—M1) of, during (Moment 2—M2), and at the end (Moment 3—M3) of the study. Students completed these measures in the classroom with support from the assistant researcher, i.e., each item was read aloud by the researcher to ensure that all students understood what was being asked. Regarding the arithmetic competency measure, data was collected at the beginning (M1) and the end (M3) of the study. 

#### 2.2.1. General Description of the Program

The Yellow’s Trials and Tribulations [35] school-based intervention was designed to promote a repertoire of SR strategies (e.g., setting-goals; time management; making decisions; monitoring tasks; self-evaluation) among fourth graders. 

#### 2.2.2. The Story-Tool Yellow’s Trials and Tribulations

The program relied on a story-tool, Yellow trials and tribulations [35] intentionally developed to convey learning strategies and promote SR processes among elementary school children. This story narrates the adventures and challenges of the rainbow colors while searching for their friend, Yellow, who is lost in the woods; the motto behind this quest is that we are all important and no one should be left behind. While reading the adventures of the rainbow colors in search for Yellow, children learn useful SR strategies likely to help them overcome difficulties and display efforts to achieve their goals. For the purpose of the current research, in each session, one chapter of the book was read aloud in class followed by a discussion of the experiences of the rainbow colors against the SR processes. These discussions helped students understand the commonalities between the experiences lived by the rainbow colors and their own, which facilitated the transfer of these learnings to students’ school life. What is more, these in class discussions were intentionally guided to provide opportunities for students to acquire, practice, and reflect on the SR strategies used by the story characters to overcome challenges and attain their purpose (i.e., find Yellow). During discussions, students were also encouraged to assign meaning to, and discover, the usefulness of the strategies learned to their school progress.

#### 2.2.3. The Self-Regulated Learning (SRL) Model

Children who SR their behavior proficiently, assume control and responsibility over their thoughts, emotions, and behaviors to achieve their self-set goals [18]. The narrative used in this intervention is grounded on the PLEE model, Planning, Execution, and Evaluation [36,37]. Rosario et. al model adds to Zimmerman’s cyclical model of the three phases a recursive loop within each phase. In the latter model, each of these phases is informed by the previous phase and informs the following phase. For example, forethought processes are informed by the self-reflection processes and inform the performance or volitional control processes. The PLEE model adds a recursive element to the previous model. For example, to complete the first phase of the SR cycle, Planning, individuals are expected to plan, execute, and evaluate their plan of action. The three phases of the cycle are set within each phase. According to Rosário and colleagues’ PLEE model (2019) the SR process presents a cyclic and a recursive nature [36,38,39]. Table 1 presents the SR strategies taught as part of the current investigation for each phase of the PLEE model.

#### 2.2.4. Session Structure

The program consisted of twelve 60-minute sessions. Throughout the program, children read and discussed the content of the 17 chapters in the story-tool. Each session began with a review of the contents worked in the previous session, followed by the reading of a new chapter. With the help of a research assistant with training in delivering SR interventions, children used the three facets of knowledge to discuss the SR processes presented in the chapter: declarative (i.e., What is? the definition of a concept), procedural (i.e., How? the operationalization of the knowledge), and conditional (When? Where? Why? in which circumstances could the knowledge be used) knowledge, e.g., [41]. 

These guidelines allowed students to assign meaning to the story and foster possible ways to apply these strategies to their daily lives. For example, one of the chapters reported the traditional story of “The Three Little Pigs”; discussion on this content provided an opportunity for students to reflect on the importance of using attention control strategies while working. Moreover, students were encouraged to examine the distractors likely to interfere in their school work and analyze strategies that may help them to avoid them. Afterwards, students were presented with a consolidation group task (e.g., make a plan for a picnic in the park with the rainbow colors). Finally, at the end of the session, children were invited to reflect on new learnings and asked to write a take-home message likely to emphasize the content discussed. 

#### 2.2.5. Treatment Integrity

Treatment fidelity procedures regarding the implementation of the protocol were as follows [42]. The three research assistants implementing the program had vast experience in delivering interventions focused on SRL. Moreover, prior to the start of the intervention, they received a dossier with session sheets detailing the protocol for each session. This dossier helped monitor the activities run in each session (e.g., the take-home message). Additionally, during the implementation of the intervention, assistant researchers met with the senior researcher every week to discuss incidents regarding the implementation and adherence to the protocol (e.g., goals set for each session). 

Finally, an expert on SRL with no participation in the implementation of the sessions watched 30% of the sessions using a protocol record sheet. Data from the video observations showed that researchers completed 91% of the activities set (range 83–95). This indicates high treatment integrity for the program.

### 2.3. Instruments and Measures

Participants were presented with a questionnaire with two parts: (i) sociodemographic information (e.g., gender, age, and the number of siblings), and (ii) instruments: one to assess SR, Inventory of Learning Self-Regulation Processes (IPAA) [43], and the other to access the SA, the subtest of the Coimbra Neuropsychological Assessment Battery (BANC) [44].

The IPAA assesses nine SRL strategies concerning the three phases of the SRL process: Planning phase comprises items 1, 3, and 7 (e.g., “I make a plan before I begin working. I think about what I want to do and how I need to complete it.”). The reability of this subscale for this study is α = 0.84, Execution phase comprises itens 2, 6, and 9 (e.g., “If I become distracted or lose concentration while I am in class or studying then I usually try to regain to achieve my goals.”). The reability of this subscale for this study is α = 0.77, and Evaluation phase comprises items 4, 5, and 8 (e.g., “I compare the grades I received with the goals I set.”). The reability of this subscale for this study is α = 0.85. The 9-item scale was rated on a five Likert scale (1, never, and 5, always). A confirmatory factorial analysis supports the construct validity of this measure (χ^2^(27, 4288)= 350.73; *p*  < 0.001; GFI = 0.982; AGFI = 0.970; TLI = 0.957; CFI = 0.968; RMSEA = 0.053, 90% CI (0.048, 0.058)).

The BANC is designed for children and adolescents from 5 to 15 years old and includes a range of subtests to evaluate different domains of neuropsychological functioning, such as memory, language, attention, executive functions, laterality, orientation, and motor function [44]. We used a performance measure to assess SA to overcome the limitation reported in the literature of using the teacher’s reports on their students’ attention in class. Literature has been alerted to that these are subjective measures that can be biased, for example, by a teacher’s expectations about their students’ performance [30]. For the purposes of this study, to assess SA, we used the Two Signal Cancellation test [44]. The reliability of this instrument in the present study was α = 0.84. The task has a duration of ten minutes, and participants are expected to perform the test individually. Each child is presented with a piece of paper with twenty-five lines of square stimuli and are asked to identify and tick the target square present at the top of the paper. This subtest allows the measurement of SA, because it requires the ability to point out two target stimuli among eight different stimuli, and sustained attention because participants are expected to maintain attention for a short period of time. 

Finally, arithmetic competency was accessed through math exercises focused on this skill (e.g., “Ana took 10€ to the camp, where she spent a week. On each of the days she was at the camp, Ana spent 0,50€. How much money did she have left?”). The 10 exercises comprising the test, and the quotation criteria for each, were developed by the eight elementary teachers. These exercises were based on the pool of exams for the fourth grade developed by the Portuguese Ministry of Education [45]. The score in each exercise varied from 0 to 2. Whenever the student presented the correct answer accompanied by an understandable explanation, it was rated with 2 points, when the student presented the correct answer but without an understandable explanation or any explanation, it was rated with 1 point, and when none of the previous answers were presented, it was rated with 0 points. The final score was obtained by adding the points of all exercises and was converted into a rating scale that varied from 1 as Insufficient to 4 as Excellent.

### 2.4. Data Analysis

The statistical analyses were run with IBM SPSS (Statistical Package for the Social Sciences), version 27.0. The analysis included (i) a descriptive analysis of data; (ii) a mixed-design analysis of variance (ANOVA) to analyze the effect of the intervention over time in SR and SA; (iii) pairwise comparisons corrected using Bonferroni adjustments to learn the differences between EG and CG and within subjects; (iv) an ANOVA unifactorial to explore the impact of SA on arithmetic competencies; and (v) a *t*-test for paired samples to analyze the arithmetic differences within subjects.

## 3. Results

Table 2 summarizes the descriptive statistical data of the three dependent variables (i.e., SRL, attention, and mathematics) for the EG and CG in the three moments. The descriptive analysis, as hypothesized, suggest that students from EG incremented their levels in all variables after the first moment. Moreover, regarding the CG, data on the three variables are inconsistent; while SR and arithmetic students presented slight fluctuations, the result in M3 was similar to that of M1. Data on SA indicates an increment over time.

### 3.1. Mixed Design ANOVA

To analyze the effect of the intervention on SR and SA over time, a mixed ANOVA was run. A significant main effect of time and group in SR (*F*(2,258) = 9.548, *p* < 0.001) and SA (*F*(2,258) = 8.766, *p* < 0.001) was found. Moreover, taking together the multivariate contrast test indicates a positive trend (Lambda Wilks = 0.381; *F*(4,514) = 79.634, *p* < 0.001, *η*^2^ = 0.383). Finally, this analysis shows an interaction between moments and group condition, which indicates that the differences between EG and CG were significant (Lambda Wilks = 0.873; *F*(4,514) = 9.035, *p* < 0.001, *η*^2^ = 0.066). This information is relevant because it clarifies the role of the intervention in explaining the differences between the assessment moments. In addition, this analysis showed that the tendency of scores is linear for both groups in SR (*F*(1, 129) = 39.951, *p* < 0.001, *η*^2^ = 0.236) and in SA (*F*(1, 129) = 271.043, *p* < 0.001, *η*^2^ = 0.678). In addition, there is a linear tendency regarding the interaction between the two groups, either in SR (*F*(1, 129) = 14.490, *p* < 0.001, *η*^2^ = 0.101) or in SA (*F*(1, 129) = 12.048, *p* < 0.001, *η*^2^ = 0.085). The pairwise comparisons for the main effect of EG and CG over the time in both variables, SR and SA, corrected using Bonferroni adjustments, are presented in Table 3 and Table 4. 

Data in Table 3 show a statistically significant effect between EG and CG, in both M2 and M3. This result is congruent with data showing that at the pre-intervention moment, no differences were found between participants in both conditions. 

Table 4 indicates that for EG, the main effect was statistically significant in the three moments for SR and SA. Data on the CG showed statistically significant differences between all moments for SA, but on SR, the data only suggest statistically significant differences between M2 and M3.

### 3.2. ANOVA Unifactorial

Acknowledging the close relationship of SA and academic achievement [13,27,28,29], particularly with arithmetic word problems; we analyzed the effect of SA on arithmetic performance. An ANOVA unifactorial was conducted to this end. The results from this analysis were summarized in Table 5. 

Results indicate that only in M3 were the differences between EG and CG (*F*(1, 134) = 18.299; *p* < 0.001, *η*^2^ = 0.070) statistically significant. These results suggest that the SR training seems to have influenced participants’ SA, with an impact on their arithmetic competencies. Building upon these results, we ran a t-test for paired samples to analyze the differences within subjects. Data indicates that the differences between M1 and M3 (*t* (67) = −3.584; *p* < 0.001) were statistically significant just for the EG.

## 4. Discussion

A school-based intervention aimed to promote SR competencies and SA among fourth-grade students was set. The design followed a quasi-experimental design with an experimental group (EG) and a control group (CG), with children randomly allocated to each condition. Extant literature has shown that the promotion of SR learning strategies empowers individuals to become active and responsible agents of their learning process [36,38,39]. To activate their agency, among other skills, individuals need to be able to focus and control their attention. For example, students who set a two-hour study session in preparation for a math test as a personal goal, need to be able to focus their attention on their studying and completion of exercises, while avoiding possible distractors [46]. In other words, students willing to attain their academic goals are expected to display SA competencies on the task and self-regulate their learning [46]. Moreover, literature has been showing that proficient SA skills are: (i) amenable to be trained, and (ii) positively associated with students’ performance in arithmetic word problems [27,29,31]. These problems are presented in a written format and require that students select the relevant information, while ignoring the irrelevant information, to come up with the computations that will help them to solve the problem [29].

This corpus of knowledge set the groundwork to the current hypotheses, suggesting that students who benefited from SR training incremented the use of SR strategies, SA, and arithmetic achievement. Current preliminary findings showed statistically significant differences between EG and CG for all the variables over time, confirming our hypotheses. These results support the actual body of evidence on the use of classroom interventions, with narratives as an effective methodology for the promotion of SR strategies [38,39] and SA.

In fact, both groups improved on their SA, which is a very interesting result. Analyzing the average results per age for the Two Signal Cancellation subtest of BANC [44], we learned that the control group presented results expected for their age group (scores between 8 and 14 points). However, students in the experimental group improved their performance showing scores expected for children aged 13/14 years old (scores between 18 and 20 points). This was an impressive and very interesting educational finding. Still, the differences between groups were found to be statistically significant, indicating a positive effect of the training on SR strategies on SA skills. Notwithstanding, future research might wish to investigate the influence of classroom variables, such as classroom routines (e.g., compositions, reading texts and drawing) in the development of this competency [46]. 

Finally, data showed that students who benefited from SR training improved their arithmetic achievement when compared to their counterparts in the CG. This finding is consistent with previous research, indicating that SR strategies, such as metacognitive monitoring, are predictors of arithmetic performance [47].

Moreover, we analyzed the effect of SA on arithmetic achievement and found positive relationships between the SA of students who benefited from the program and their arithmetic achievement. These results confirm the proximal relationship between SA and arithmetic achievement competency, as reported by previous research [27,28,29]. In sum, despite preliminary findings, current results are important to help educators and school administrators reflect on the need for the use of effective strategies to promote SR and attentional control competencies in school settings. As extant research reports, students with training in SR and SA competence are likely to improve their agency, use learning strategies to help them complete school tasks, and focus their efforts on attaining self-set academic goals [21,29]. We believe current research addressed the reported gap in the literature in this area [9,10,11], and adds literature by showing a successful path to improve SA competencies in elementary school. 

Moreover, the results from this study support important implications for educational practice. For example, educators and school administrators could consider using narrative-based programs for promoting SR learning strategies and SA in elementary schools. Moreover, and acknowledging prior data showing that many elementary school students struggle to focus their attention on school tasks [10], school administrators could consider organizing training for teachers on SR and SA competencies to help them deliver and train this strategic content in class. In fact, teachers could consider embedding the “Yellow Trial and Tribulations” educational tool [35] within class content to train students in bettering their learning strategies and SA.

Some limitations in this study should be acknowledged. The first regards the use of self-report as a single measure to assess SR. This is a limitation, because self-report measures may not be sensitive enough to capture the actual effects of the intervention. Future studies should consider complementing the assessment protocol with performance measures (e.g., Hanoi Tower) and other sources of information (e.g., teachers and/or parents/legal guardians’ perceptions). Moreover, according to Stevens and Bavelier [29] the effect of SA on the performance of mathematics word problems is mediated by working memory. However, working memory was not assessed in the current study. Future research may wish to analyze the effect of SR training on students working memory. Lastly, future research might find it interesting to collect follow-up data (e.g., 6 months after the intervention) to understand whether the positive effects found would be maintained over time.

To conclude, the current data, despite preliminary data, encourages researchers and educators’ effort to organize training on SR and SA, in order to promote students’ competencies and arithmetic achievement.

## Figures and Tables

**Table 1:** SR learning strategies for each phase of the PLEE model (adapted from Zimmerman and Martinez-Pons (1986) [40]).

Planning	Self-Evaluation Goal-setting and planning Enviromental structuring Seeking social assistance
Execution	Organization and Transformation Seeking information Keeping records and monitoring Rehearsing and memorizing
Evaluation	Reviewing records Self-consequences

**Table 2:** Descriptive Statistics for SR, SA and Arithmetic dependent variables.

	Control Group		Experimental Group	
	N = 68		N = 68	
	M	SD	M	SD
Self-regulation				
M1	3.59	0.76	3.65	0.63
M2	3.39	1.03	3.95	0.63
M3	3.57	1.05	4.19	0.55
Selective Attention				
M1	6.75	10.09	6.67	6.66
M2	11.58	7.43	14.68	5.06
M3	14.48	8.29	19.27	5.61
Arithmetic				
M1	2.57	1.1	2.74	0.7
M3	2.57	0.87	3.01	0.74

Note. M = Mean; SD = Standard Deviation, M1: moment 1; M2: moment 2; M3: moment 3.

**Table 3:** Bonferroni’s adjusted *p* values for all possible pairwise differences among two dependent variables of the between-subjects factor.

	Control Group vs. Experimental Group		
	Moment 1	Moment 2	Moment 3
	Mean Difference	Mean Difference	Mean Difference
Self-Regulation	−0.03	−0.46 **	−043 ***
Selective Attention	0.12	−3.49 **	−4.51 ***

Note. ** *p < 0.01,* *** *p < 0.001*. M1: moment 1; M2: moment 2; M3: moment 3.

**Table 4:** Bonferroni’s adjusted *p* values for all possible pairwise differences among two dependent variables of the within-subjects factor.

	Control Group	Experimental Group
	Mean Difference	Mean Difference
Self-Regulation		
M1–M2	0.13	−0.30 ***
M2–M3	−0.26 **	−0.23 *
M1–M3	−0.13	−0.53 ***
Selective Attention		
M1–M2	−4.64 ***	−8.01 ***
M2–M3	−3.58 ***	−4.59 ***
M1–M3	−8.21 ***	−12.60 ***

Note. * *p <* 0.05, ** *p <* 0.01, *** *p <* 0.001. M1: moment 1; M2: moment 2; M3: moment 3.

**Table 5:** Analysis of Variance (ANOVA) unifactorial for arithmetic competency.

	Control Group vs. Experimental Group					
	SS	df	MS	*F*	*p*	*η* ^2^
Arithmetic						
M1	0.89	1	0.82	1.05	0.308	0.01
M3	6.62	1	6.62	10.12	0.002 **	0.07

Note. SS: Square Sum; df: degree of freedom; Mean Square; *F*: effect size; *η*^2^: eta squared ** *p* < 0.01. M1: moment 1; M2: moment 2; M3: moment 3.
